# The bladder’s turn: first-in-human combined bladder–kidney transplantation

**DOI:** 10.3389/ti.2026.17955

**Published:** 2026-10-28

**Authors:** Emilien Seizilles de Mazancourt, Oriol Bestard

**Affiliations:** 1 Paris Institute for Transplantation and Organ Regeneration (PITOR), Université Paris Cité, INSERM U970 PARCC, Paris, France; 2 Department of Urology, Hôpital Saint-Louis, AP-HP, Paris, France; 3 Nephrology and Kidney Transplantation Department, Vall d’Hebron University Hospital, Barcelona, Spain

**Keywords:** bladder transplantation, combined kidney transplantation, neurogenic bladder, urinary reconstruction, vascularized composite allograft

## Introduction

In June 2026, Nima Nassiri and Inderbir Gill reported in *The Lancet* the first bladder transplantation performed in a living human, carried out at University of California Los Angeles (UCLA) and University of Southern California (USC) as a combined vascularized composite bladder allograft (VCBA) and kidney transplant within a prospective first-in-human feasibility trial [1, 2]. We warmly congratulate the authors and their team. This result did not come out of nowhere. It is the product of a deliberate, stepwise programme that moved from cadaveric feasibility work [3] to porcine models, perfused human cadavers and robotic bladder auto-transplantation in heart-beating brain-dead research donors [4, 5], each step published and open to scrutiny. It is a model of how surgical innovation should be translated to the clinic.

## A forgotten, then mistreated organ

The bladder has long been the poor relation of transplantation. When Mathieu Jaboulay performed his pioneering kidney grafts to the vessels at the elbow in 1906, the question of urinary drainage barely arose [6]. The lower urinary tract was simply left out of the picture. Ironically, decades later, the bladder was enlisted as a convenient outlet for the exocrine secretions of pancreas grafts. Bladder drainage in simultaneous pancreas-kidney transplantation came at the price of chemical cystitis, haematuria, urethritis, recurrent infections and metabolic acidosis, and it was largely abandoned in favour of enteric drainage [7, 8]. Yet, the bladder is far from a passive bystander in kidney transplantation. For a small but particularly challenging group of patients with severe lower urinary tract dysfunction or an absent bladder, providing a safe and durable urinary reservoir remains a major and often underappreciated clinical problem driving morbidity and progressive graft dysfunction. Although reconstructive procedures and urinary diversion have enabled transplantation in many such patients, they carry their own substantial burden of complications and may fall short of restoring a functional lower urinary tract. First forgotten, then mistreated, the bladder has at last become the transplanted organ itself.

## The first case

The recipient was a 41-year-old man on peritoneal dialysis. He had undergone bilateral nephrectomy for bilateral, low-risk clear-cell renal cell carcinoma treated more than 5 years earlier, and a partial cystectomy for urachal adenocarcinoma [1]. Pretransplant urodynamics showed a small-capacity bladder with storage pressures above 40 cmH_2_O and vesicoureteral reflux. Donor bladder capacity and compliance were checked during procurement by gravity filling with 350 mL of saline, with intraluminal pressure staying below 30 cmH_2_O. A back-table cystoscopy excluded mucosal abnormalities. As in the preclinical work [5], the VCBA was based on both internal iliac pedicles, conjoined into a single arterial and a single venous channel, an elegant solution reminiscent of the Y-graft reconstruction familiar to pancreas transplant surgeons. In the recipient, the cystectomy was completed while preserving the bladder neck and a rim of trigone. The transplant ureter was reimplanted into the allograft bladder, and a suprapubic catheter was left in place. The whole procedure took about 8 h.

The postoperative course was not uneventful. On day 25, an intraperitoneal urine leak from the suprapubic tube tract, with wound breakdown, required reoperation (Clavien–Dindo grade IV) [1]. This complication does not call into question the technical feasibility of the procedure, and urinary leaks are a recognized complication of any bladder or ureterovesical reconstruction [9]. It nonetheless remains relevant to the assessment of safety and should inform the refinement of perioperative management, notably urinary drainage and wound care, in recipients who often have a previously operated abdomen and pelvis. At 6 months, kidney function was stable (glomerular filtration rate 52–55 mL/min per 1.73 m^2^). Protocol VCBA biopsies at days 35, 100 and 180 showed no cellular or antibody-mediated rejection, and donor-derived cell-free DNA stayed at very low levels, reaching 0.04% at 6 months.

The most striking result is function. The trial’s primary endpoint was technical feasibility. Functional success was modestly defined as the ability to store more than 200 mL, and candidates had to be able to self-catheterize, since the allograft was expected to behave as a denervated reservoir [1, 4]. Instead, at 6 months the patient had intact bladder sensation, a capacity of 600 mL with normal compliance and no detrusor overactivity, and he voided to completion with a maximum flow rate of 17 mL/s, negligible post-void residuals and full continence. The authors candidly acknowledge that they did not expect spontaneous voiding. They propose intrinsic myogenic activity, local stretch responses, abdominal straining and a trigonal contribution as plausible but unproven explanations [1]. Whether this is reproducible is now the central question, and the first information on subsequent recipients calls for caution. According to an institutional report from UCLA Health dated 23 June 2026, which has not been peer reviewed, two further patients have since received a combined transplant: in one, the bladder allograft had to be removed after a complication while the kidney graft continued to function; the other was voiding without a catheter almost 2 months after surgery [10]. These preliminary observations must be distinguished from the peer-reviewed data, and we eagerly await their formal reporting.

## Why not bowel, or a bioengineered bladder?

The current standard for patients with an end-stage bladder is reconstruction with intestine, through augmentation enterocystoplasty, a continent reservoir or a non-continent ileal conduit. These procedures work, but their costs are well known: hyperchloraemic metabolic acidosis, vitamin B_12_ deficiency and bone loss, mucus production, stones, chronic bacteriuria and recurrent infection, anastomotic strictures and a lifelong need for surveillance [11]. In kidney transplant recipients, colonised bowel segments combined with immunosuppression add a further layer of infectious risk to the graft.

Tissue engineering once seemed to offer a way out. Atala and colleagues reported in 2006 autologous urothelial and smooth-muscle cells seeded on a collagen scaffold for augmentation cystoplasty in seven children with myelomeningocele [12]. Omental wrapping improved results, compliance and capacity increased, and there was no mucus production. However, a phase II study using a similar approach failed to reproduce these results and reported serious adverse events [13]. The limitations are now better understood: insufficient neovascularisation, fibrosis and graft shrinkage, and the toxicity of urine for an unprotected regenerating wall [11]. Bladder organoids and assembloids are a remarkable tool for studying regeneration and cancer [14], but they are still a long way from a full-thickness, vascularized and compliant reservoir. This is the core argument for a vascularized allograft: it brings its own blood supply and its own mature urothelial barrier.

Conversely, the vascularized allograft provides a valuable biological reference for regenerative approaches, because it shows what a functional bladder substitute must achieve. Beyond adequate perfusion and an intact urothelial barrier, compliant storage depends on the architecture of the extracellular matrix, the organization of the smooth-muscle bundles and the way the tissue remodels over time; it is possibly the disruption of these features by fibrosis that has undermined engineered grafts. Serial analysis of transplanted bladders, for example, of the collagen-to-smooth-muscle ratio, elastin content and fibrosis, could help define the structural targets that bioengineered constructs need to reproduce. Transplantation and regenerative medicine should therefore be seen as complementary rather than competing approaches.

## The immunosuppression trade-off, revisited

The objection most often raised is that bladder transplantation simply “trades” the morbidity of intestinal segments for lifelong immunosuppression [15]. This is true for an isolated bladder transplant. It is much less true for a combined kidney–bladder transplant, where immunosuppression is required anyway for the kidney. The reasoning is the same one that justified simultaneous pancreas-kidney transplantation decades ago. In the case reported, no particularly intensive regimen appears to have been needed to protect the bladder allograft [1]. An existing indication for immunosuppression does not, however, exclude graft-related risks: the bladder allograft can itself be rejected, adds its own surgical morbidity, and may fail and require removal, as illustrated by one of the subsequent cases [10].

There is also encouraging, though indirect, precedent regarding the immunogenicity of bladder tissue. Every kidney transplant already carries donor urothelium, in the renal pelvis and ureter, and decades of experience have not revealed a specific urothelial rejection syndrome or an excess of donor-derived ureteral tumours. More specifically, donor bladder tissue has been transplanted for years in *en bloc* pediatric kidney transplantation using a bladder patch [16–19], including in our own experience in Lyon [20]. The aim of the patch is to avoid ureterovesical reimplantation of very small ureters, where the risk of stenosis is high given their calibre. These grafts have been managed with standard kidney transplant immunosuppression. These observations are reassuring, but they do not establish the immunogenicity of a complete vascularized bladder allograft, which contains a far larger mass of detrusor, vessels and connective tissue than a ureter or a bladder patch.

## Not a solution for bladder cancer

Urothelial carcinoma is by far the leading indication for cystectomy, which raises the obvious question of oncological candidates. In a letter about the brain-dead autotransplantation model, Davis and colleagues were concerned that bladder transplantation would have to be timed with cystectomy [21]. In their reply, Nassiri and Gill made clear that the target population is not bladder cancer but primarily neurogenic and other non-oncological end-stage bladders [22]. We fully agree, and would add two points. First, the timing is fundamentally different: the indication and timing of an oncological cystectomy must never be modified by the prospect of a transplant. Second, many patients with recent urothelial carcinoma would in any case be contraindicated for kidney transplantation until an adequate cancer-free interval has passed. Consistently, the UCLA/USC protocol excludes donors with a history of urothelial or prostate malignancy [1], although recipients with a remote, low-risk oncological history are not excluded outright, as the first patient illustrates. Some have suggested that already immunosuppressed transplant recipients with localized or high-risk non-muscle-invasive disease might 1 day be considered [11]. That scenario should remain strictly within trials.

## Selecting the right recipient and the right donor

Patient selection will largely determine success. A small bladder in a dialysis patient is not necessarily an end-stage bladder. Inoue and colleagues showed urodynamically that defunctionalised, hypocompliant bladders recover capacity and compliance once diuresis is restored by a kidney graft [23]. Pretransplant bladder cycling can also help separate reversible defunctionalisation from fixed neurogenic or fibrotic disease, avoiding unnecessary augmentation [24]. A thorough pretransplant work-up, including urodynamics, cystoscopy and imaging of the lower urinary tract [25], together with a cycling trial where appropriate, should be required before any candidate is judged to have a “terminal” bladder. The patients who stand to gain most are those in whom end-stage renal disease and end-stage bladder coexist, often because lower urinary tract dysfunction caused the renal failure in the first place [11].

Two further points deserve attention. First, the preserved bladder neck and trigonal rim have probably contributed to the excellent functional result. Outcomes may therefore not apply to patients with an incompetent or absent outlet, such as those with exstrophy or after radical cystoprostatectomy. Second, donor assessment remains a real challenge. Intraoperative filling and back-table cystoscopy are sensible, but cystoscopy performed in the donor before procurement, together with a detailed urological history, would allow unsuitable bladders to be excluded before the organ is committed.

Logistics also matter. The preclinical programme relied on robotic procurement and autotransplantation [4, 5], which raised understandable questions about feasibility within a multi-organ procurement. The Lancet report shows that the procedure can be done without the robot [1]. Coordination with the other procurement teams will nonetheless need to be planned, since the donor iliac vessels are also used as vascular grafts for liver and pancreas transplantation.

## A unique model that should be fully exploited

The heart-beating brain-dead donor model was first developed for xenotransplantation, when pig-to-non-human primate studies could no longer answer the questions that stood in the way of clinical translation [26, 27]. The UCLA/USC team was right to borrow it for bladder transplantation [4]. The xenotransplantation experience also teaches a second lesson. Each of these rare cases should be studied as deeply as possible, with the kind of multimodal phenotyping (histology, transcriptomics, spatial and single-cell approaches, and circulating biomarkers) that has transformed our understanding of the first pig-to-human kidney xenografts [28].

The bladder lends itself particularly well to such an approach. It can be biopsied by cystoscopy with little morbidity. It is continuously bathed in urine, which makes urinary donor-derived cell-free DNA and urinary transcriptomic signatures natural companions to the plasma assay used in this trial. In a combined kidney–bladder transplant, the easily accessible bladder could even serve as a sentinel for kidney rejection, a reversal of the situation in pancreas–kidney transplantation. VCBA will also need shared histological criteria for rejection, as has been done for other vascularized composite allografts. Functional mechanisms deserve the same effort. Repeated urodynamics with detrusor pressure recordings and sphincter electromyography, together with sensory and neurophysiological testing, should help determine whether true detrusor contractions and reinnervation occur, as suggested in rat models [29], or whether voiding relies on other mechanisms. Longitudinal assessment of graft perfusion, for example, by Doppler or contrast-enhanced ultrasound, and of fibrosis and smooth-muscle integrity on serial biopsies, correlated with urodynamic parameters, would help clarify the relationship between graft structure and function. Until reinnervation is directly demonstrated, spontaneous voiding should be regarded as a functional observation rather than as evidence of reinnervation.

## Potential indications today

On the basis of current evidence [1, 5, 11], candidates for bladder transplantation could include: (i) patients with end-stage renal disease and an end-stage bladder, in whom a combined kidney-bladder transplant avoids adding immunosuppression, particularly when lower urinary tract dysfunction caused the kidney failure; (ii) kidney transplant recipients already on immunosuppression who develop a terminal bladder; and (iii) patients with severe neurogenic bladder, chronic inflammatory bladder disease or refractory, recurrent infections who are not suitable for, or have failed, intestinal reconstruction.

## Conclusion

Nassiri, Gill and their team have turned a long-standing idea into a clinical reality through careful and transparent preparation. A single peer-reviewed case cannot establish efficacy, and the subsequent recipients, followed with rigorous urodynamic, immunological and histological monitoring, will tell us whether the unexpected return of voiding is the rule or the exception, and how often graft-related complications occur. We look forward to these results, and to the international collaboration that such a rare and demanding procedure will require. After more than a century on the sidelines of transplantation, the bladder has finally had its turn.

## Data Availability

The original contributions presented in the study are included in the article/supplementary material, further inquiries can be directed to the corresponding author.
